# Quantitative Analysis of Smooth Pursuit and Saccadic Eye Movements in Multiple Sclerosis

**DOI:** 10.3390/neurolint18020022

**Published:** 2026-01-26

**Authors:** Pavol Skacik, Lucia Kotulova, Ema Kantorova, Egon Kurca, Stefan Sivak

**Affiliations:** 1 Neurology Department, University Hospital Martin, Kollarova 2, 036 01 Martin, Slovakia; 2 Jessenius Faculty of Medicine Martin, Comenius University Bratislava, Mala Hora 4, 036 01 Martin, Slovakia; 3 Biomedical Centre Martin, Jessenius Faculty of Medicine Martin, Comenius University Bratislava, 036 01 Martin, Slovakia

**Keywords:** multiple sclerosis, eye tracking, oculomotor dysfunction, smooth pursuit, saccades

## Abstract

**Introduction**: Multiple sclerosis (MS) is a chronic inflammatory and neurodegenerative disease of the central nervous system, frequently associated with visual and oculomotor disturbances. Quantitative analysis of eye movements represents a non-invasive method for assessing central nervous system dysfunction beyond conventional imaging; however, the diagnostic and predictive value of oculomotor metrics remains insufficiently defined. Objectives: The aims of this study were to compare smooth pursuit gain and reflexive saccade parameters (latency, velocity, and precision) between individuals with MS and healthy controls, and to evaluate their ability to discriminate disease status. **Methods**: This cross-sectional study included 46 clinically stable patients with MS (EDSS ≤ 6.5) and 46 age- and sex-matched healthy controls. Oculomotor function was assessed using videonystagmography under standardized conditions. Group differences across horizontal and vertical gaze directions were analyzed using linear mixed-effects models. Random forest models were applied to assess the discriminative performance of oculomotor parameters, with permutation-based feature importance and receiver operating characteristic (ROC) curve analysis. **Results**: Patients with MS showed significantly reduced smooth pursuit gain across most horizontal and vertical directions compared with controls. Saccadic latency was significantly prolonged in all tested movement directions. Saccadic velocity exhibited selective directional impairment consistent with subtle medial longitudinal fasciculus involvement, whereas saccadic precision did not differ significantly between groups. A random forest model combining pursuit and saccadic parameters demonstrated only moderate discriminative performance between MS patients and controls (AUC = 0.694), with saccadic latency contributing most strongly to classification. **Conclusions**: Quantitative eye-movement assessment revealed widespread oculomotor abnormalities in MS, particularly reduced smooth pursuit gain and prolonged saccadic latency. Although the overall discriminative accuracy of oculomotor parameters was limited, these findings support their potential role as complementary markers of central nervous system dysfunction. Further longitudinal and multimodal studies are required to clarify their clinical relevance and prognostic value.

## 1. Introduction

Multiple sclerosis (MS) is a chronic inflammatory and neurodegenerative disease of the central nervous system (CNS), characterised by multifocal lesions in white and grey matter that variably affect the brain, spinal cord, and optic pathways [1]. Early and accurate diagnosis is crucial for initiating disease-modifying therapies (DMTs), which improve long-term outcomes when started at the earliest stages of the disease [2].

In recent decades, the systematic analysis of eye-movement behaviour has emerged as a promising approach for neurological diagnosis, disease monitoring, and rehabilitation [3]. Abnormalities of smooth pursuit and saccadic eye movements are common in MS and may indicate central nervous system involvement extending beyond regions detectable on conventional MRI [4,5,6].

Eye-movement assessments are non-invasive, readily accessible, and compatible with technologies such as video nystagmography (VNG), eye-tracking systems, and virtual reality (VR)-based testing. These methods provide high-resolution, objective data on central neurological control [7,8]. Emerging evidence suggests that oculomotor alterations may contribute to evaluating treatment response or identifying prodromal stages of MS [9,10,11].

Accordingly, the present study was designed as an exploratory investigation aimed at characterising oculomotor differences between individuals with MS and healthy controls, rather than as a confirmatory study intended to establish diagnostic biomarkers. Specifically, this study assessed whether smooth pursuit gain and reflexive saccadic parameters (latency, velocity, and precision) differed between individuals with MS and healthy controls, and they were explored for their potential diagnostic value for disease classification in a hypothesis-generating, cross-sectional context.

## 2. Patients and Methods

The study was approved by the Ethics Committee of the Jessenius Faculty of Medicine, Comenius University, in Bratislava. Written informed consent was obtained from all participants prior to inclusion. Patients with definite MS according to the 2017 McDonald criteria, aged 18–55 years, and with Expanded Disability Status Scale (EDSS) scores ≤ 6.5 were recruited from the MS Centre, University Hospital Martin, Slovakia. All patients were clinically stable at the time of testing, with no relapse or corticosteroid treatment within the preceding three months. None of the patients exhibited severe scotomas or marked reductions in visual acuity on routine clinical examination prior to assessment. Exclusion criteria included the presence of other central or peripheral vestibular disorders, severe comorbid neurological or ophthalmological disease and without contraindication to oculomotor testing. Clinical disability was assessed by a neurologist certified in EDSS evaluation.

A total of 46 patients diagnosed with MS were included in the study, consisting of 25 women and 21 men, with an average age of 39 years (95% CI: 32–45 years). The clinical phenotype distribution comprised 65.2% relapsing–remitting MS, 21.8% clinically isolated syndrome, 8.7% secondary progressive MS, and 4.3% primary progressive MS. Patients were stratified according to EDSS into three subgroups: MS1: EDSS ≤ 1.5 (n = 19), MS2: EDSS > 1.5 to ≤ 4.0 (n = 16), MS3: EDSS > 4.0 to ≤ 6.5 (n = 11).

A control group of 46 healthy participants, matched to the MS group for age (±5 years) and sex, was also recruited. Control subjects had no history of multiple sclerosis, other central nervous system disorders, or peripheral/central vestibular disease, and none had contraindications to oculomotor testing.

## 3. Examination Protocol

All patients first underwent a standard clinical neurological and vestibular examination. Oculomotor assessment was then performed using VNG with the SYNAPSYS VNG ULMER system (SYNAPSYS, Marseille, France). Saccadic and smooth pursuit eye movements were evaluated using the VISIO mask system (SYNAPSYS, Marseille, France), which allows simultaneous binocular tracking.

Participants were seated in a fixed chair facing a projection screen displaying visual stimuli consisting of a white square on a black background. After receiving standardised instructions, the VISIO mask was applied and calibrated through randomised saccadic stimulation of varying direction, amplitude, and frequency.

Saccadic testing was conducted in both horizontal and vertical planes with stimulation parameters of 30° amplitude and 0.4 Hz frequency. Examination of eye movements, including saccades and smooth pursuit, was performed for each eye in both horizontal and vertical directions: right eye right (RER), right eye left (REL), left eye right (LER), left eye left (LEL), right eye up (REU), right eye down (RED), left eye up (LEU), and left eye down (LED). Smooth pursuit was examined under identical stimulation conditions. Average gain values for smooth pursuit and average saccadic parameters (latency, peak velocity and accuracy) were recorded.

## 4. Statistical Analysis

All data were analyzed using the R programming language (version 4.4.2). Prior to building the classification models, exploratory data analysis was performed. For continuous variables, boxplots were constructed; for categorical variables, mosaic plots were used. Random forest classification models (500 trees; terminal nodes limited to a single observation) were used to assess the ability of oculomotor indicators (saccade- and pursuit-related metrics) to discriminate between predefined groups (case vs. control and MS1 vs. MS2 vs. MS3) within the study sample. Given the cross-sectional design, these analyses were intended solely to evaluate within-sample classification. Feature importance was assessed via the permutation method. For each model, an ROC curve was constructed using out-of-bag (OOB) data, with a 95% confidence interval estimated by bootstrap. Depending on the model, predictors included gain, latency, velocity, and precision—either individually or combined with clinical variables (age, disease duration, sex, and MS phenotype)—and classification performance was evaluated using ROC curves.

Smooth pursuit and saccade outcomes (all directions) were analyzed using linear mixed-effects models with a random intercept for subject: response ~ sex + age + group × direction + (1|sample_ID). Outcomes included pursuit gain and saccade latency, velocity, and precision; in case-only analyses (MS1–MS3), disease duration and disease type were additionally included.

Model assumptions were evaluated using exploratory plots (histograms, boxplots, Q–Q plots) and residual diagnostics (DHARMa quantile residuals and standard residual checks). When normality was violated, transformations were selected using powerTransform() (car package) with rounded λ, supported by symbox() (car package). When extreme observations persisted, extreme values were flagged using identify_outliers() (rstatix package) and excluded (≤5 subjects per model; exact exclusion counts for each outcome model are provided in Supplementary Table S3), and the same model was refitted with and without flagged values; the version with superior residual diagnostics and overall fit was retained. Estimated marginal means were computed using emmeans, with Tukey-adjusted pairwise comparisons; back-transformation was applied when relevant.

Supplementary direction-specific Wilcoxon rank-sum tests were performed as sensitivity analyses requested during peer review. Because covariate adjustment and within-subject correlation were not accommodated, these results were not used for primary inference relative to the mixed-effects models. *p*-values were adjusted using the Benjamini–Hochberg false discovery rate procedure, and results are reported in Supplementary Table S2.

## 5. Results

### 5.1. Group Comparisons of Smooth Pursuit Gain

Based on the constructed linear mixed-effects models, estimated marginal means of the pursuit gain parameter were calculated for the right and left eyeballs, across all movement directions—rightward and leftward in the horizontal plane, and upward and downward in the vertical plane. These estimates were then compared pairwise between group levels within each horizontal and vertical direction. The group levels included case, control, and MS1–MS3 as specified by the model.

**Figure 1:**
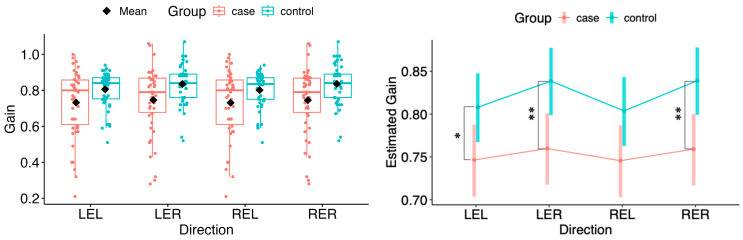
(**Left**): Boxplots of Gain by group and horizontal directions. (**Right**): EMMs with 95% CIs. Contrasts: *p*  <  0.05 (*), *p*  <  0.01 (**). LEL, left eye looking left; LER, left eye looking right; REL, right eye looking left; RER, right eye looking right.

Comparisons of gain between cases and controls showed significant differences in right-eye (RER) and left-eye right (LER) direction and the left-eye left (LEL) direction (LEL: Δ = −0.062, 95% CI −0.120 to −0.0034, *p* = 0.038; LER: Δ = −0.079, 95% CI −0.136 to −0.0215, *p* = 0.0075; RER: Δ = −0.080, 95% CI −0.137 to −0.0226, *p* = 0.0067). The right-eye left (REL) direction contrast was borderline at the 5% level (REL: Δ = −0.058, 95% CI −0.116 to 0.00014, *p* = 0.0506) (see Figure 1). For readability, the complete set of model-based contrasts with 95% confidence intervals and *p*-values across all directions/eyes is provided in Supplementary Table S1.

**Figure 2:**
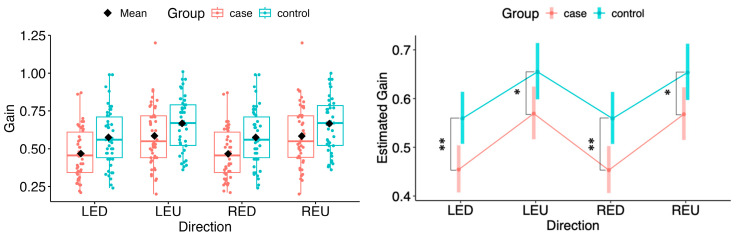
(**Left**): Boxplots of Gain by group and vertical directions. (**Right**): EMMs with 95% CIs. Contrasts: *p*  <  0.05 (*), *p*  <  0.01 (**). LED, left eye looking down; LEU, left eye looking up; RED, right eye looking down; REU, right eye looking up.

In case of vertical directions, all movements with both eyes revealed significant differences between the case and control groups (LED: Δ = −0.105, 95% CI −0.177 to −0.033, *p* = 0.0047; LEU: Δ = −0.086, 95% CI −0.165 to −0.0065, *p* = 0.0341; RED: Δ = −0.106, 95% CI −0.178 to −0.034, *p* = 0.0043; REU: Δ = −0.086, 95% CI −0.165 to −0.0066, *p* = 0.0339), consistent with the box-plot visualisations (see Figure 2).

**Figure 3:**
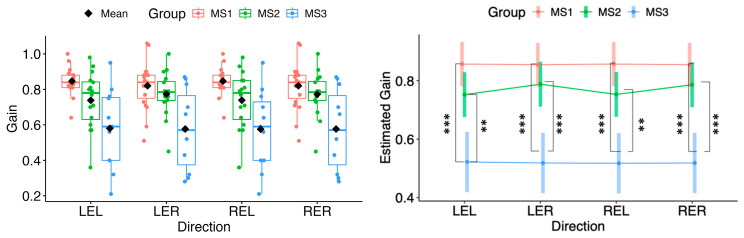
(**Left**): Boxplots of Gain by MS group and horizontal directions. (**Right**): EMMs with 95% CIs. Contrasts: *p*  <  0.01 (**), *p*  <  0.001 (***). LEL, left eye looking left; LER, left eye looking right; REL, right eye looking left; RER, right eye looking right.

As shown in Figure 3, post hoc pairwise comparisons found no significant difference between MS1 and MS2 in any horizontal direction. In contrast, MS1 differed from MS3 in all directions (LEL *p* = 3.6 × 10^−5^; LER *p* = 3.3 × 10^−5^; REL *p* = 2.9 × 10^−5^; RER *p* = 3.3 × 10^−5^). MS2 differed from MS3 in all directions (LEL *p* = 0.00270; LER *p* = 0.000430; REL *p* = 0.00213; RER *p* = 0.000471).

**Figure 4:**
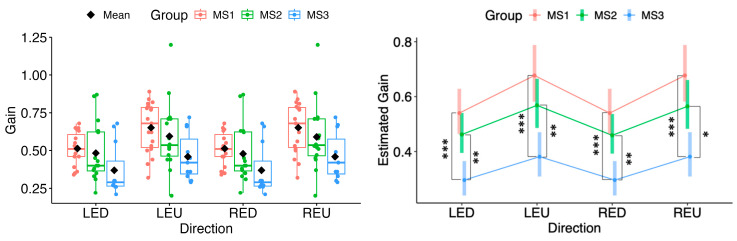
(**Left**): Boxplots of Gain by MS group and vertical directions. (**Right**): EMMs with 95% CIs. Contrasts: *p*  <  0.05 (*), *p*  <  0.01 (**), *p*  <  0.001 (***). LED, left eye looking down; LEU, left eye looking up; RED, right eye looking down; REU, right eye looking up.

In the vertical directions, none of the MS1–MS2 contrasts were statistically significant; however, MS3, in pairwise comparisons with MS1 and MS2, showed significantly different estimates of marginal means across all vertical directions (MS1 vs. MS3: LED *p* = 1.24 × 10^−4^; LEU *p* = 2.36 × 10^−4^; RED *p* = 1.24 × 10^−4^; REU *p* = 2.36 × 10^−4^; MS2 vs. MS3: LED *p* = 0.00330; LEU *p* = 0.00902; RED *p* = 0.00383; REU *p* = 0.01034) (see Figure 4).

### 5.2. Random Forest Classification Using Smooth Pursuit Gain

**Figure 5:**
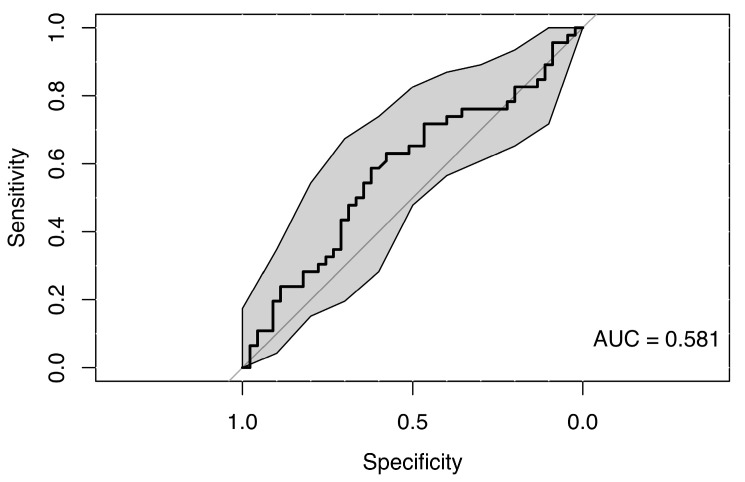
ROC Curve (with 95% CI) Using Smooth Pursuit and Clinical Parameters for Group Classification.

The use of a random forest classification model incorporating predictors such as gain values across all eye movement directions, sex, and age did not reveal any clear differences between the MS group and healthy controls, suggesting a lack of strong association. The resulting weak ROC curve (AUC = 0.581) confirmed the model’s low ability to distinguish between cases and controls (see Figure 5).

**Figure 6:**
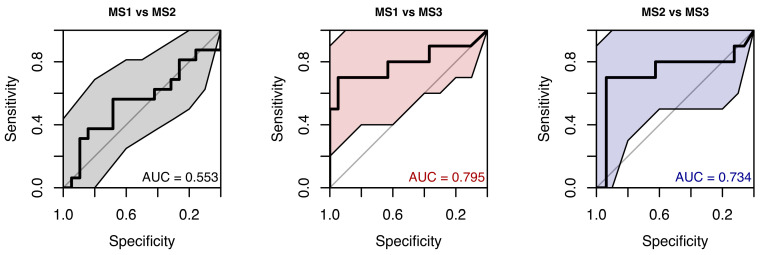
ROC Curves (with 95% CIs) Using Only Smooth Pursuit Parameters for MS Group Classification.

The random forest classification model for the MS1, MS2, and MS3 subgroups, based on gain parameters from all eye movement directions, produced ROC curves with AUCs ranging from 0.55 to 0.79. The distinction between MS3 and the other subgroups may appear clearer; however, given the small number of observations within each MS level (10–19), the estimates are likely unstable, which is also reflected by the wide confidence intervals (see Figure 6).

### 5.3. Group Comparisons of Saccades

#### 5.3.1. Latency

**Figure 7:**
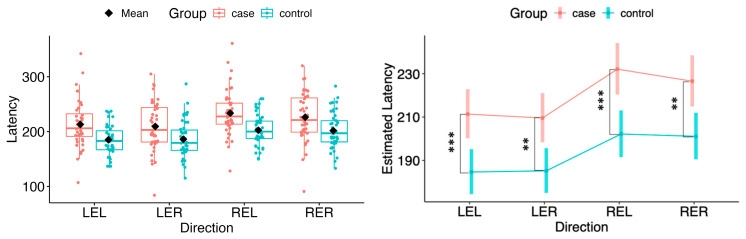
(**Left**): Boxplots of Latency by group and horizontal directions. (**Right**): EMMs with 95% CIs. Contrasts: *p*  <  0.01 (**), *p*  <  0.001 (***). LEL, left eye looking left; LER, left eye looking right; REL, right eye looking left; RER, right eye looking right.

**Figure 8:**
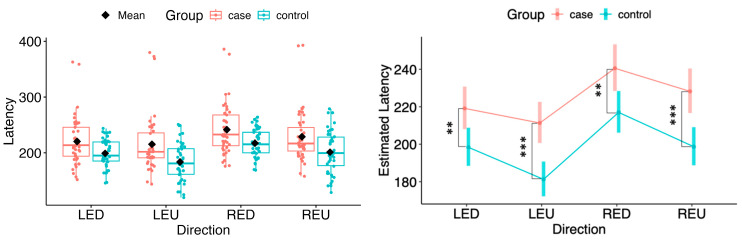
(**Left**): Boxplots of Latency by group and vertical directions. (**Right**): EMMs with 95% CIs. Contrasts: *p* <  0.01 (**), *p*  <  0.001 (***). LED, left eye looking down; LEU, left eye looking up; RED, right eye looking down; REU, right eye looking up.

In both horizontal and vertical gaze directions, pairwise testing between cases and controls revealed a statistically significant difference in the estimated marginal means of saccadic latency for both eyes across all tested movement directions (LEL: Δ = 26.71, 95% CI 11.30 to 42.12, *p* = 0.000822; LER: Δ = 24.38, 95% CI 9.04 to 39.73, *p* = 0.00209; REL: Δ = 29.95, 95% CI 13.89 to 46.02, *p* = 0.000337; RER: Δ = 25.44, 95% CI 9.47 to 41.42, *p* = 0.00203; LED: Δ = 20.76, 95% CI 5.53 to 35.99, *p* = 0.00795; LEU: Δ = 30.09, 95% CI 15.71 to 44.47, *p* = 6.29 × 10^−5^; RED: Δ = 23.55, 95% CI 6.85 to 40.24, *p* = 0.00606; REU: Δ = 29.54, 95% CI 13.88 to 45.20, *p* = 0.000285) (see Figure 7 and Figure 8).

**Figure 9:**
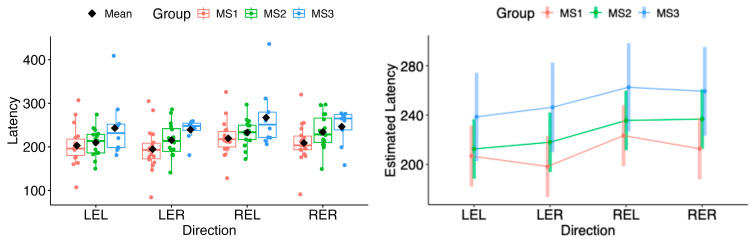
(**Left**): Boxplots of Latency by MS group and horizontal directions. (**Right**): EMMs with 95% CIs. LEL, left eye looking left; LER, left eye looking right; REL, right eye looking left; RER, right eye looking right.

**Figure 10:**
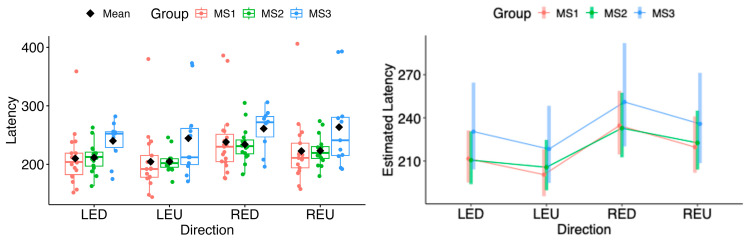
(**Left**): Boxplots of Latency by MS group and vertical directions. (**Right**): EMMs with 95% CIs. LED, left eye looking down; LEU, left eye looking up; RED, right eye looking down; REU, right eye looking up.

In comparisons among the MS subgroups (MS1, MS2, MS3), the model-based estimated marginal means of saccadic latency did not differ significantly in any movement direction (all *p* > 0.05) (see Figure 9 and Figure 10).

As sensitivity analyses, direction-specific Wilcoxon rank-sum comparisons for gain and latency are reported in Supplementary Table S2; none remained significant after Benjamini–Hochberg FDR correction.

##### Random Forest Classification Using Latency

**Figure 11:**
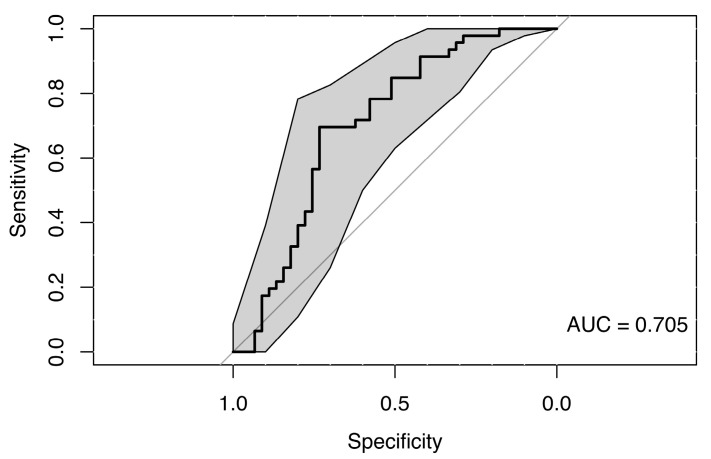
ROC Curve (with 95% CI) Using Saccades Latencies and Clinical Parameters for Group Classification.

The use of a random forest classification model incorporating saccadic latency values across all eye movement directions, along with sex and age as predictors, did not reveal any clear differences between the MS group and healthy controls, suggesting a lack of strong association. The resulting weak ROC curve (AUC = 0.705) confirmed the model’s low discriminative ability to distinguish between cases and controls (see Figure 11).

**Figure 12:**
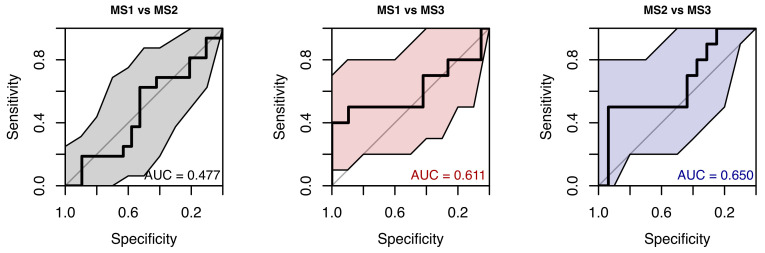
ROC Curves (with 95% CIs) Using Only Saccades Latencies Parameters for MS Group Classification.

When using only saccadic latency parameters, the model’s ability to distinguish between MS subgroups was pretty weak (AUC 0.48–0.65) (see Figure 12). Given the modest subgroup sizes and the weak discrimination, additionally non-parametric pairwise subgroup comparisons were performed as a sensitivity analysis (Supplementary Table S2).

#### 5.3.2. Velocity

**Figure 13:**
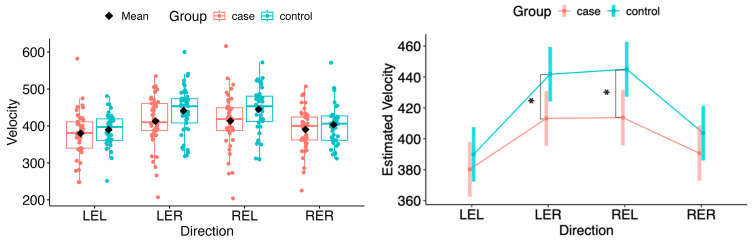
(**Left**): Boxplots of Velocity by group and horizontal directions. (**Right**): EMMs with 95% CIs. Contrasts: *p*  <  0.05 (*). LEL, left eye looking left; LER, left eye looking right; REL, right eye looking left; RER, right eye looking right.

**Figure 14:**
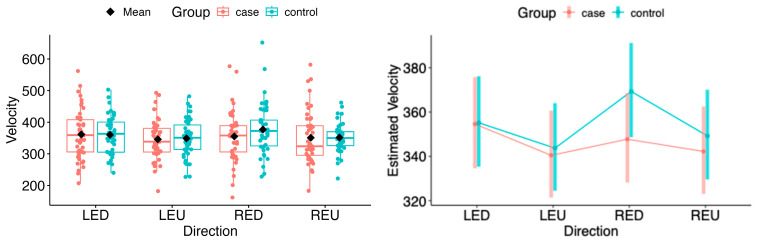
(**Left**): Boxplots of Velocity by group and vertical directions. (**Right**): EMMs with 95% CIs. LED, left eye looking down; LEU, left eye looking up; RED, right eye looking down; REU, right eye looking up.

For the estimated marginal means of saccadic velocity, a statistically significant deviation was observed in the horizontal plane during rightward movement of the left eye (LER: Δ = −28.58, 95% CI −53.64 to −3.52, *p* = 0.0265) and leftward movement of the right eye (REL: Δ = −31.27, 95% CI −56.53 to −6.01, *p* = 0.0156) (see Figure 13 and Figure 14).

In the vertical plane, no statistical difference in velocity was found between the MS group and controls (see Figure 14).

**Figure 15:**
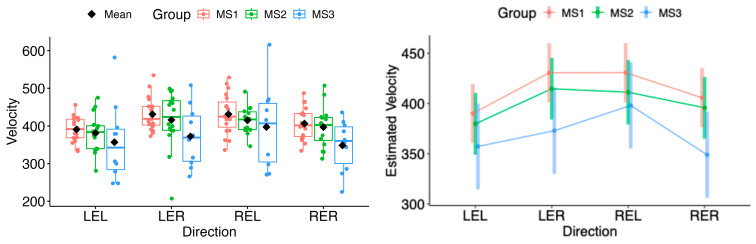
(**Left**): Boxplots of Velocity by MS group and horizontal directions. (**Right**): EMMs with 95% CIs. LEL, left eye looking left; LER, left eye looking right; REL, right eye looking left; RER, right eye looking right.

**Figure 16:**
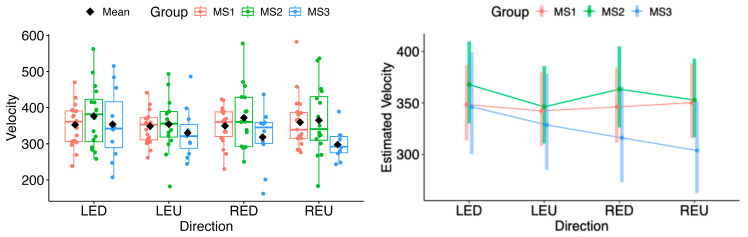
(**Left**): Boxplots of Velocity by MS group and vertical directions. (**Right**): EMMs with 95% CIs. Contrasts: LED, left eye looking down; LEU, left eye looking up; RED, right eye looking down; REU, right eye looking up.

In the subgroup analysis, the estimated marginal means of velocity in both horizontal and vertical directions did not differ significantly for either eye (see Figure 15 and Figure 16).

##### Random Forest Classification Using Velocity

**Figure 17:**
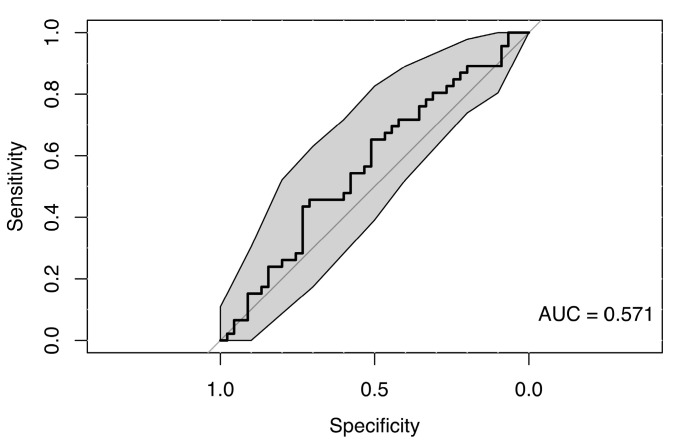
ROC Curve (with 95% CI) Using Saccade Velocity and Clinical Parameters for Group Classification.

The random forest classification model using saccadic velocity values across all eye movement directions, along with sex and age as predictors, did not reveal any clear differences between the MS group and healthy controls, suggesting a lack of strong association. The resulting weak ROC curve (AUC = 0.571) confirmed the model’s low ability to distinguish between cases and controls (see Figure 17).

**Figure 18:**
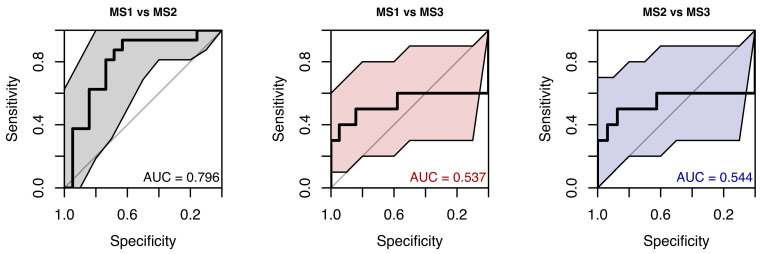
ROC Curves (with 95% CIs) Using Only Saccade Velocity Parameters for MS Group Classification.

Random forest models were trained to classify pairwise combinations of MS1–MS3 subgroups using saccadic velocity. Performance was similarly poor to the model distinguishing patients from controls. Discriminative ability among MS subgroups was limited, with ROC AUCs from 0.537 to 0.796. Wide confidence intervals persisted because of the small number of observations in each MS subgroup (see Figure 18).

#### 5.3.3. Precision

**Figure 19:**
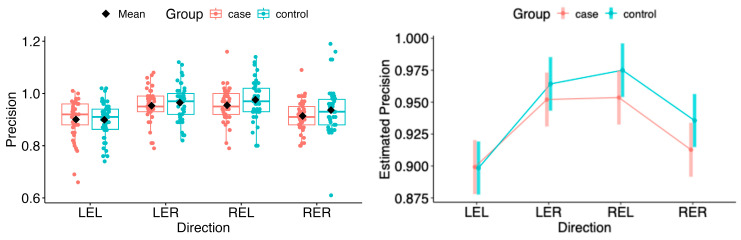
(**Left**): Boxplots of Precision by group and horizontal directions. (**Right**): EMMs with 95% CIs. LEL, left eye looking left; LER, left eye looking right; REL, right eye looking left; RER, right eye looking right.

**Figure 20:**
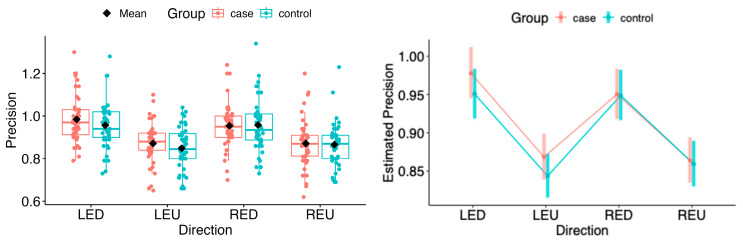
(**Left**): Boxplots of Precision by group and vertical directions. (**Right**): EMMs with 95% CIs. LED, left eye looking down; LEU, left eye looking up; RED, right eye looking down; REU, right eye looking up.

The point estimates of the marginal means for saccadic precision showed no statistically significant differences between the patient group and controls in the vertical and horizontal directions (see Figure 19 and Figure 20).

**Figure 21:**
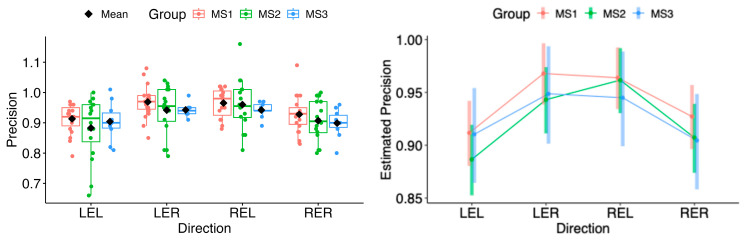
(**Left**): Boxplots of Precision by MS group and horizontal directions. (**Right**): EMMs with 95% CIs. LEL, left eye looking left; LER, left eye looking right; REL, right eye looking left; RER, right eye looking right.

**Figure 22:**
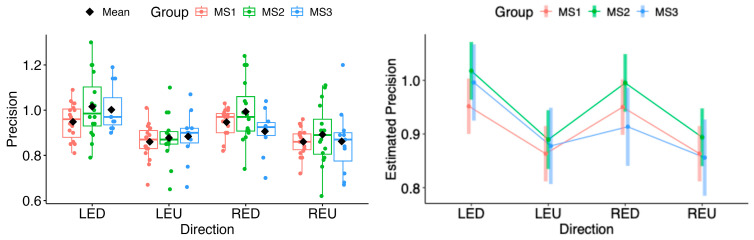
(**Left**): Boxplots of Precision by MS group and vertical directions. (**Right**): EMMs with 95% CIs. LED, left eye looking down; LEU, left eye looking up; RED, right eye looking down; REU, right eye looking up.

The same situation persisted in pairwise comparisons of estimated marginal means among MS1–MS3. No statistical differences were found in the horizontal or vertical movement directions (see Figure 21 and Figure 22).

##### Random Forest Classification Using Precision

**Figure 23:**
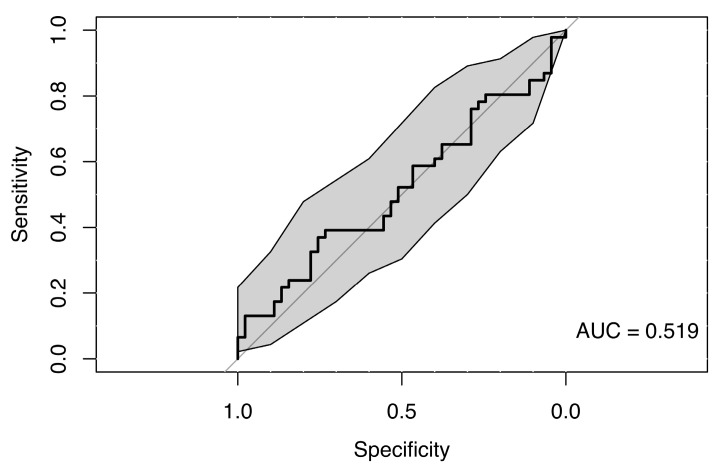
ROC Curve (with 95% CI) Using Saccades Precision and Clinical Parameters for Group Classification.

The random forest classification model using precision values across all eye movement directions, along with sex and age as predictors, did not reveal any clear differences between the MS group and healthy controls, indicating a lack of strong association. The resulting weak ROC curve (AUC = 0.519) confirmed the model’s poor ability to distinguish between cases and controls (see Figure 23).

**Figure 24:**
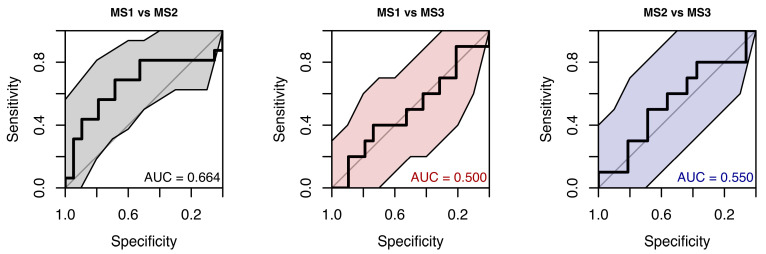
ROC Curves (with 95% CIs) Using Only Saccades Precision Parameters for MS Group Classification.

Performance of the random forest model that used only saccadic precision to classify samples into MS subgroups was also only slightly above chance. ROC curves showed AUCs from 0.50 (chance) to 0.664, with wide CIs (see Figure 24).

**Figure 25:**
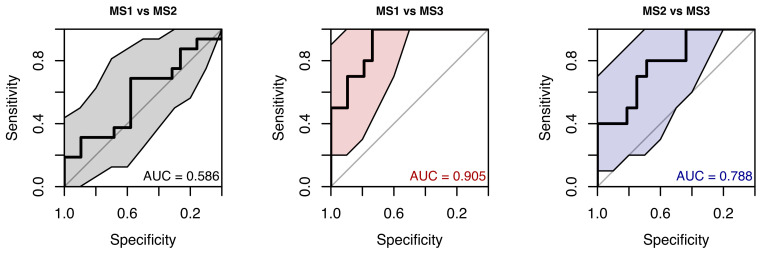
ROC Curves (with 95% CIs) Using Saccades Precision and Clinical Parameters for MS Group Classification.

The random forest classification model, augmented with clinical predictors, still performed poorly. ROC AUCs ranged from 0.586 to 0.905, and confidence intervals remained wide (see Figure 25).

### 5.4. Random Forest Classification Using Oculomotor Parameters

A random forest model including all smooth-pursuit and saccade parameters was fitted to evaluate overall discrimination power. This model differs from the separate models (with latencies, precisions, velocities, and gains analyzed individually) in that variables were assessed jointly rather than in isolation. The model was constructed to determine how well all parameters together could distinguish cases from controls.

**Figure 26:**
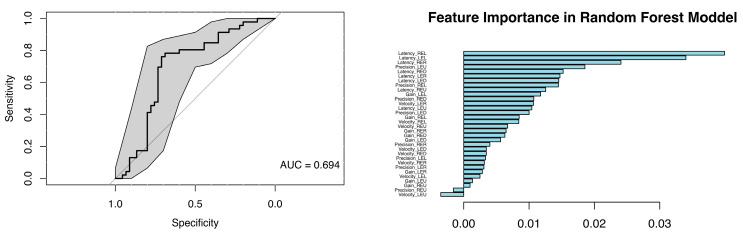
(**Left)**: ROC Curve (with 95% CIs) from a RF Model Using all Saccades and Smooth Pursuit Parameters to Classify into Group. (**Right**): Feature Importance scores from the Same Model.

The resulting ROC curve showed an AUC of 0.694, which is interpreted as only moderate discrimination. Thus, these parameters are not strong biomarkers for case–control classification. According to the feature-importance scores, the highest contributions were consistently assigned to latency measures across several directions (see Figure 26).

**Figure 27:**
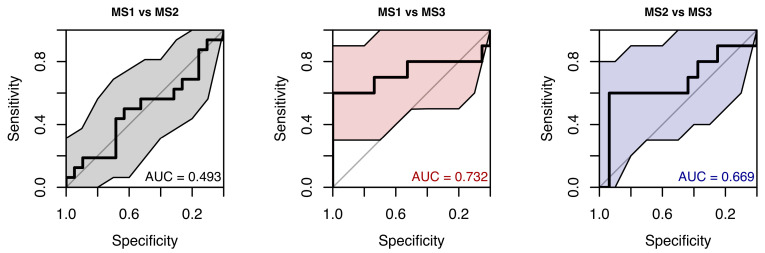
ROC Curves (with 95% CIs) from an RF Model Using all Saccades and Smooth Pursuit Parameters to Classify Samples into MS Group.

In a random forest model classifying samples into MS groups using all available saccade and smooth-pursuit parameters, the resulting ROC curves showed weak performance, with AUCs ranging from 0.50 to 0.73 (see Figure 27).

Subgroup findings should be interpreted with caution, as the MS1–MS3 analyses were exploratory and underpowered, limiting the reliability of subgroup-specific inferences.

## 6. Discussion

Abnormalities in smooth pursuit and saccadic eye movements have been documented in patients with multiple sclerosis (MS) [12,13]. In this cross-sectional study, we quantitatively evaluated smooth pursuit and saccadic eye movements using video nystagmography (VNG). The cohort included patients across a spectrum of disability levels, as well as an age- and sex-matched healthy control group. We analysed key oculomotor parameters—pursuit gain and saccadic latency, velocity, and precision—across horizontal and vertical gaze directions.

Statistically significant reductions in gain parameters across most directions suggest involvement of the smooth pursuit system in patients with MS. Similar differences have been reported previously [14].

Given that smooth pursuit relies on a distributed cortico-cerebellar network, including extrastriate motion-processing areas, pontine nuclei, and cerebellar floccular structures, this system may be particularly susceptible to the diffuse white matter and cerebellar pathology characteristic of MS [4,15]. The presence of pursuit deficits in patients with minimal clinical disability supports the notion that smooth pursuit dysfunction may reflect subclinical network disruption rather than being solely a consequence of advanced disease [16]. These abnormalities may therefore be detectable even in patients with low Expanded Disability Status Scale (EDSS) scores, suggesting early dysfunction. Furthermore, smooth pursuit impairment has been observed during the initial phases of MS [5,17]. Pursuit dysfunction tends to increase with higher EDSS scores and has been associated with greater disability, consistent with reports of progressive oculomotor impairment in MS [18].

Among directional effects, vertical gain was most affected, particularly during downward pursuit. This pattern may suggest that vertical gaze centres are more vulnerable to MS-related damage; however, this interpretation should be made with caution, as lower gain values are generally observed in the vertical plane even under physiological conditions [19]. The observed up–down asymmetry could reflect bilateral floccular involvement, with preferential impairment of downward pursuit, potentially related to asymmetries in vertical gaze-velocity Purkinje cells within the flocculus [20]. Importantly, although some degree of vertical disadvantage and directional asymmetry is also present in healthy controls [21], the generally and statistically significantly lower gain values observed in the MS group—most pronounced in vertical directions—support the view that MS likely acts as a contributing factor, even if these abnormalities may partially overlap with normal inter-individual variability.

Visual exploration of natural scenes is strongly influenced by environmental structure and task demands, which are often organised along the horizontal axis and may therefore favour horizontal scanning strategies [22]. This bias may reflect inherent physiological differences between horizontal and vertical pursuit systems, as vertical smooth pursuit typically exhibits lower gain and different dynamic properties across both healthy adult and paediatric populations. However, these differences should be interpreted as normative characteristics of oculomotor organisation rather than evidence of directional dominance [23,24].

Significantly prolonged saccadic latencies were observed in patients with MS compared with healthy controls across all movement directions and in both eyes. Such latency prolongation likely reflects the complex neuroanatomy underlying saccade generation, which depends on coordinated activity across visual pathways as well as cortical, subcortical, and brainstem structures, including frontal oculomotor and attentional networks [25,26]. Additional contributing factors may include impairments in attentional control and executive processing [27,28], fatigue [29], and structural neurodegenerative changes such as brain atrophy and T1 “black holes” [30]. Although a trend towards longer latencies with increasing EDSS was observed, subgroup comparisons did not reach statistical significance, possibly reflecting the modest sample size or variability within subgroups.

Saccadic velocity was reduced in patients with MS compared with healthy controls. Several factors may contribute to the observed velocity reduction, including disease severity, lesion burden, and fatigue [25,26,27,28,29]. Impaired neuronal connectivity within the central nervous system, particularly between brainstem and cerebellar structures, has also been associated with disturbances in saccadic velocity [29]. Statistically significant differences were observed in the left-eye rightward (LER) and right-eye leftward (REL) directions. This pattern is consistent with adduction impairment associated with mild dysfunction of the medial longitudinal fasciculus (MLF). These findings may reflect subclinical or partial internuclear ophthalmoplegia, which may not be evident on bedside examination but can be detected using quantitative video-oculography, underscoring the sensitivity of eye-tracking techniques for identifying early or mild MLF involvement [31,32]. Moreover, as the MLF has been linked to central fatigue, oculomotor changes related to its dysfunction may provide diagnostic and symptomatic insight, as well as potential markers of treatment response [33]. Taken together, these mechanisms suggest that saccadic slowing reflects a multifactorial manifestation of distributed neural network dysfunction rather than isolated oculomotor pathway damage [34].

In our data, saccadic precision showed no significant reduction compared with healthy controls, indicating that it may be the least sensitive saccadic parameter. This finding is consistent with previous reports [27]. Although its discriminative value appears limited, precision measures may nonetheless complement pursuit and saccadic parameters in comprehensive oculomotor assessment, particularly for identifying subtle cerebellar or adaptive changes during disease progression.

The random forest model combining all smooth pursuit and saccadic parameters achieved only moderate discrimination between patients with MS and healthy controls (AUC = 0.694). Feature-importance analysis identified saccadic latency as the strongest contributor; however, even this parameter demonstrated limited discriminative power. The modest performance of the random forest models indicates that oculomotor parameters alone are unlikely to serve as standalone biomarkers for MS. Eye-movement metrics may therefore be most informative when interpreted alongside neuroimaging, electrophysiological, and cognitive measures, as multidisciplinary approaches are increasingly recognised to provide a more comprehensive characterisation of oculomotor dysfunction and its relationship with cognitive and neural network changes in MS [10].

## 7. Conclusions

Patients with MS in our study group exhibited significant alterations in smooth pursuit and saccadic eye-movement parameters, consistent with involvement of distributed central nervous system networks. Although within-sample classification analyses demonstrated only moderate performance, quantitative eye-tracking may represent a useful non-invasive approach for characterising oculomotor dysfunction.

### Study Limitations and Future Directions

The cross-sectional design and modest sample size limit causal inference and generalisability. Disease-modifying therapy status, fatigue, and MRI findings were not assessed, and the timing of testing in relation to fatigue and medication intake was not standardised. These unmeasured factors may have influenced eye-movement outcomes. Future longitudinal and multimodal studies are warranted to further clarify the clinical and neural relevance of these findings.

## Data Availability

The original contributions presented in this study are included in the article. Further inquiries can be directed to the corresponding author.

## Supplementary Materials

The following supporting information can be downloaded at: https://www.mdpi.com/article/10.3390/neurolint18020022/s1, Table S1: Smooth pursuit and saccade parameters measured in all directions, providing case–control comparisons and MS1, MS2, and MS3 group data with estimated effects, standard errors (SE), confidence limits (CL), and *p*-values; Table S2: Pairwise Wilcoxon *p*-values and Benjamini–Hochberg–adjusted q-values for oculomotor parameters across MS1, MS2, and MS3 groups; Table S3: Linear mixed-model outcomes for case–control and case-only analyses, including patients excluded from gain, latency, velocity, and precision analyses.
