# Supplementary material for: Quantitative Analysis of Smooth Pursuit and Saccadic Eye Movements in Multiple Sclerosis
Source: Neurol Int. 2026 Jan 26;18(2):22. doi: 10.3390/neurolint18020022 (PMC12943389; doi:10.3390/neurolint18020022)
Supplement: Supplementary file 1 [file neurolint-18-00022-s001.zip › Supplementary_Table_S2.html]

| **Characteristic** | **MS1**  N = 191 | **MS2**  N = 161 | **MS3**  N = 101 | Pairwise Wilcoxon p-values | | | BH-adjusted q-values | | |
| --- | --- | --- | --- | --- | --- | --- | --- | --- | --- |
|  |  |  |  | **p MS2 vs MS1** | **p MS3 vs MS1** | **p MS3 vs MS2** | **q MS2 vs MS1** | **q MS3 vs MS1** | **q MS3 vs MS2** |
| Latency\_RER | 204 (193, 242) | 229 (209, 269) | 267 (239, 276) | 0.085 | 0.029 | 0.225 | 0.204 | 0.159 | 0.311 |
| Latency\_LER | 194 (171, 229) | 214 (187, 244) | 248 (225, 258) | 0.233 | 0.057 | 0.147 | 0.311 | 0.172 | 0.261 |
| Latency\_REL | 218 (196, 239) | 234 (216, 251) | 251 (221, 280) | 0.289 | 0.057 | 0.257 | 0.339 | 0.172 | 0.325 |
| Latency\_LEL | 196 (176, 224) | 214 (186, 229) | 232 (198, 253) | 0.619 | 0.183 | 0.187 | 0.661 | 0.300 | 0.300 |
| Latency\_REU | 216 (194, 255) | 220 (209, 232) | 233 (214, 279) | 0.804 | 0.347 | 0.280 | 0.804 | 0.387 | 0.336 |
| Latency\_LEU | 193 (178, 239) | 204 (195, 210) | 212 (197, 257) | 0.540 | 0.313 | 0.246 | 0.589 | 0.357 | 0.319 |
| Latency\_RED | 230 (204, 257) | 231 (217, 243) | 272 (239, 283) | 0.778 | 0.033 | 0.073 | 0.795 | 0.159 | 0.185 |
| Latency\_LED | 205 (180, 238) | 213 (196, 223) | 253 (223, 256) | 0.655 | 0.035 | 0.040 | 0.683 | 0.159 | 0.159 |
| Gain\_RER | 0.84 (0.73, 0.88) | 0.79 (0.74, 0.85) | 0.62 (0.43, 0.83) | 0.208 | 0.009 | 0.061 | 0.303 | 0.099 | 0.172 |
| Gain\_LER | 0.84 (0.73, 0.88) | 0.79 (0.74, 0.85) | 0.62 (0.43, 0.83) | 0.267 | 0.009 | 0.058 | 0.328 | 0.099 | 0.172 |
| Gain\_REL | 0.83 (0.81, 0.88) | 0.78 (0.62, 0.85) | 0.62 (0.40, 0.76) | 0.108 | 0.005 | 0.091 | 0.207 | 0.099 | 0.207 |
| Gain\_LEL | 0.83 (0.81, 0.88) | 0.78 (0.62, 0.85) | 0.62 (0.40, 0.76) | 0.104 | 0.005 | 0.108 | 0.207 | 0.099 | 0.207 |
| Gain\_REU | 0.68 (0.51, 0.79) | 0.54 (0.46, 0.71) | 0.44 (0.36, 0.66) | 0.208 | 0.012 | 0.120 | 0.303 | 0.099 | 0.221 |
| Gain\_LEU | 0.68 (0.51, 0.79) | 0.54 (0.46, 0.71) | 0.44 (0.36, 0.66) | 0.233 | 0.012 | 0.102 | 0.311 | 0.099 | 0.207 |
| Gain\_RED | 0.51 (0.46, 0.63) | 0.40 (0.36, 0.63) | 0.30 (0.27, 0.56) | 0.154 | 0.039 | 0.069 | 0.264 | 0.159 | 0.183 |
| Gain\_LED | 0.51 (0.46, 0.63) | 0.40 (0.36, 0.63) | 0.30 (0.27, 0.56) | 0.202 | 0.039 | 0.054 | 0.303 | 0.159 | 0.172 |
|  |  |  |  |  |  |  |  |  |  |
| --- | --- | --- | --- | --- | --- | --- | --- | --- | --- |
| 1 Median (Q1, Q3) | | | | | | | | | |
